# Consumer Assessment of Healthcare Providers and Systems Among Racial and Ethnic Minority Patients With Alzheimer Disease and Related Dementias

**DOI:** 10.1001/jamanetworkopen.2022.33436

**Published:** 2022-09-27

**Authors:** Asmaa Albaroudi, Jie Chen

**Affiliations:** 1 Department of Health Policy and Management, School of Public Health, University of Maryland, College Park; 2 The Hospital And Public Health Interdisciplinary Research Lab, School of Public Health, University of Maryland, College Park

## Abstract

**Importance:**

Consumer Assessment of Healthcare Providers and Systems (CAHPS) measures have been used widely to measure patient-centered care. Evidence is needed to understand CAHPS measures among racial and ethnic minority patients with Alzheimer disease and related dementias (ADRD).

**Objective:**

To examine racial and ethnic disparities in CAHPS among patients with ADRD and to examine the association between social determinants of health and CAHPS disparities.

**Design, Setting, and Participants:**

This study focused on patients with ADRD who were enrolled in Medicare Shared Savings Program Accountable Care Organizations (ACOs). The primary data sets were the 2017 Medicare Beneficiary Summary File and the beneficiary-level ACO data. The study population was limited to community-based beneficiaries who had a diagnosis of ADRD and were aged 65 years and older. Cross-sectional analyses and the decomposition approach were implemented. Data were analyzed from November 2021 to July 2022.

**Exposure:**

Enrollment in a Medicare Shared Savings Program ACO.

**Main Outcomes and Measures:**

Six ACO CAHPS measures were included: getting timely care, appointments, and information; how well providers communicate; patients’ rating of provider; access to specialists; health promotion and education; and shared decision-making. ACO CAHPS were continuous measures with possible ranges from 0 to 100. The summation of these 6 measures as an overall index was also created. In CAHPS measures, the term *provider* can include hospitals, home health care agencies, and doctors, among others.

**Results:**

The final sample included 568 368 beneficiaries (347 783 female patients [61.2%]; 38 030 African American patients [6.69%], 6258 Asian patients [1.10%], 18 231 Hispanic patients [3.21%], and 505 849 White patients [89.0%]; mean [SD] age, 82.17 [7.95] years). Significant racial and ethnic disparities in CAHPS scores were observed. After controlling for beneficiary, hospital, and area characteristics, compared with their White counterparts, African American or Black (coefficient = −1.05; 95% CI, −1.15 to −0.95; *P* < .001), Asian (coefficient = −0.414; 95% CI, −0.623 to −0.205; *P* < .001), and Hispanic (coefficient = −0.099; 95% CI, −0.229 to 0.032; *P* = .14) patients with ADRD reported lower total CAHPS scores. Disparities were also observed among individual ACO CAHPS. Decomposition results showed that a proxy for social determinants of health explained 10% to 13% of disparities of ACO CAHPS between African American or Black vs White and Hispanic vs White patients with ADRD. Most of the racial and ethnic disparities, especially those between White and Asian individuals, could not be explained by the models used to analyze data.

**Conclusions and Relevance:**

These results demonstrated significant variations in CAHPS by race and ethnicity among patients with ADRD enrolled in ACOs. Social determinants of health are critical in explaining racial and ethnic disparities. More research is needed to explain disparities in CAHPS.

## Key Points

**Question:**

Do disparities in Consumer Assessment of Healthcare Providers and Systems (CAHPS) exist between racial and ethnic groups with Alzheimer disease and related dementias (ADRD)?

**Findings:**

In this cross-sectional study, significant racial and ethnic disparities in CAHPS score were observed according to the analysis of 568 368 community-based Medicare beneficiaries aged 65 years and older who were enrolled in an Accountable Care Organization. A proxy for social determinants of health explained 10% to 13% of CAHPS disparities.

**Meaning:**

These findings suggest that it is imperative to implement targeted efforts to decrease disparities that could result in improvements in timely care and communication with clinicians for the aging diverse population with ADRD.

## Teaser

This cross-sectional study evaluates racial and ethnic disparities in Consumer Assessement of Healthcare Providers and Systems measures among patients with Alzheimer Disease and related dementias.

## Introduction

It is estimated that there are more than 5 million US residents with Alzheimer disease and related dementias (ADRD) and that this number will increase to 13.9 million US residents by 2060.^1^ The incidence of dementia among Black and Hispanic older adults is approximately double and 1.5 times that of White older adults, respectively.^2^ The variation in ADRD risk is partly associated with differences in health conditions and socioeconomic status, among other factors for Black and Hispanic older adults relative to their White counterparts.^3,4^ Evaluation of patients’ (and their caregivers’) satisfaction and communication with health care clinicians is critical, especially for people with ADRD. For example, a patient’s trust in a clinician is linked with improved health behaviors.^4^ Often, health care clinicians develop this trust via communication (eg, relayed clear explanation, being an active listener), demonstrating care (eg, exhibiting intent to address their health condition), and showing competence (eg, having expertise, being methodical).^5^

Consumer Assessment of Healthcare Providers and Systems (CAHPS) measures have been used widely in the literature to measure this patient- centered, consumer-centered care through the patient experience. CAHPS surveys patient-physician communication, patient’s self-reported satisfaction with the clinicians, and shared decision-making. Lower CAHPS scores often reflected lack of health care access and lack of patient trust, and members of racial and ethnic minority groups were more likely to report poor patient experience (ie, lower CAHPS scores).^6,7,8,9,10^ CAHPS measures related to access to specialists, communication, educational information, timeliness, and shared decision-making are all substantial factors in the care experience of patients with ADRD and their caregivers.^11^

To our knowledge, this is the first study to examine CAHPS measures among patients with ADRD and associated racial and ethnic disparities. We hypothesized that patients with ADRD belonging to racial and ethnic minority groups, on average, scored lower on CAHPS than their White counterparts. Social determinants of health (SDOH) have been constantly identified as one of the main barriers to quality health care.^12^ Physicians caring for Hispanic patients found challenges in delivering high-quality care for several reasons, including obstacles with communication.^13^ Patients with limited English ability may encounter barriers when attempting to engage.^14^ Separately, factors such as cultural preferences may impact the ideal care setting for persons with ADRD.^15^ Hence, we further hypothesized that the differences between CAHPS by race and ethnicity, if any, might reflect variations of SDOH and cultural preferences.

## Methods

This cross-sectional analysis followed the Strengthening the Reporting of Observational Studies in Epidemiology (STROBE) reporting guideline. The study was approved by the University of Maryland institutional review board. The requirement for informed consent was waived because the study used secondary data analysis. Our study focused on patients with ADRD who were enrolled in Medicare Shared Savings Program (MSSP) Accountable Care Organizations (ACOs). Approximately 37% of Medicare enrollees with ADRD participated in an ACO in 2017.^16^ Beneficiaries receiving care from ACOs, relative to non-ACO enrollees, had better experiences with access to care and care coordination.^17^ Our study focused on this fairly homogeneous population given the data availability, and we speculated that the racial and ethnic disparities might be more pronounced among the entire aging ADRD population.

### Data

According to the National Institute on Minority Health and Health Disparities framework, our study included measures at multilevels. The primary data sets of the study were the 2017 Centers for Medicare and Medicaid Services Medicare Beneficiary Summary File (CMS MBSF). To capture ACO characteristics, we further linked the CMS MBSF with the 2017 MSSP ACO data. Specifically, using the ACO identifiers, we were able to capture the ACO CAHPS evaluations reported by the CMS 2017 ACO Quality Reporting Documentation.^18^ Finally, we merged our data with the 2017 American Community Survey and the Area Health Resource File using beneficiaries’ zip codes and county identifications.

### Population

Our study focused on patients with a diagnosis of ADRD using the CMS Chronic Conditions Data Warehouse measure. The sample was limited to community-based fee-for-service (at least 12 months) beneficiaries aged 65 years and older. Medicare Advantage and dual-eligible beneficiaries were excluded from the analysis. Patients were linked to the ACO beneficiary level file shared savings program.

### Dependent Variables

Our study focused on ACO CAHPS measures endorsed by the National Quality Forum. The CAHPS survey assesses the outcome of ACOs on patient as well as caregiver experiences. Specifically, ACO CAHPS includes, but is not limited to, an analysis of the patient’s experience with care coordination, use of specialists, shared decision-making, and health education. CAHPS survey data can be found on the Care Compare and are used by the Quality Payment Program.^18^

In the main results, we report our findings using CAHPS indicators as continuous variables to capture the variations in the scores. Six ACO CAHPS measures were included: Getting Timely Care, Appointments, and Information (ACO1); How Well Your Providers Communicate (ACO2); Patients’ Rating of Provider (ACO3); Access to Specialists (ACO4); Health Promotion and Education (ACO5); and Shared Decision-Making (ACO6). ACO1 through ACO6 were continuous measures with possible ranges from 0 to 100. We created the summation of these 6 measures as an overall index.

### Independent Variables

The key independent variable in this study was the Research Triangle Institute Race Code. The variable includes the following racial and ethnic groups: African American or Black (Black), Asian or Pacific Islander (Asian), Hispanic or Latinx (Hispanic), and non-Hispanic White (White). We included several covariates, controlling for age, sex, and health. We also accounted for a range of health indicators, common coexisting chronic conditions for ADRD, obtained from the MBSF using the Chronic Conditions Data Warehouse: acute myocardial infarction, asthma, atrial fibrillation, heart failure, chronic obstructive pulmonary disease, depression, diabetes, hyperlipidemia, hypertension, and stroke or transient ischemic attack.^19^

We included SDOH measures in the analysis. Consistent with the literature on ADRD, we included zip code level percentages of residents with high school degrees and higher, living in poverty, and who were African American or Black.^20^ We also included the indicator of whether the whole county was a mental health shortage area. State indicators were included in the main analysis. Alternatively, we also used the Area Deprivation Index (ADI) as an overall indicator of the SDOH of the area.^21^ ADI allows for rankings of neighborhoods by socioeconomic disadvantages in a region, considering the domains of income, education, employment, and housing quality. ADI has been used to inform health policy design for disadvantaged neighborhood groups.^22^ We created an index if the ADI score of beneficiaries’ zip codes was higher than the ADI national rank of 50%, indicating persons residing in socioeconomically challenged neighborhoods.

### Statistical Analysis

Analysis for this study was at the beneficiary level. We first compared the ACO CAHPS measures, characteristics of beneficiaries, and SDOH by race and ethnicity. We used the ordinary least square regression to estimate the variation of ACO CAHPS measures by race and ethnicity. We first reported the regression results only controlling for beneficiaries’ demographic and health indicators (model 1). Then, we expanded model 1 by applying the state-fixed effect ordinary least square estimation and controlling for measures of SDOH (model 2). We applied the full model (model 2) to examine the overall ACO CAHPS (the summation) score as well as each individual ACO CAHPS score (ACO1-ACO6).

We were interested in understanding whether our models with the beneficiary and area-level measures could explain racial and ethnic disparities in ACO CAHPS. To do this, we applied the Blinder-Oaxaca decomposition model.^23,24,25,26^ The Blinder-Oaxaca decomposition model is a regression-based model. For example, to decompose the difference in the ACO CAHPS score between Black and White patients with ADRD, ordinary least square regressions of these 2 groups were estimated separately. These 2 estimations were then rearranged to indicate (1) the portion of the difference due to observed characteristics (ie, all of the control variables), and (2) the portion of the difference due to unobserved heterogeneities, such as cultural background and discrimination. Among the observed population characteristics, disparities associated with each specific factor, such as SDOH, were quantified. Similar approaches were applied to study the disparities of ACO CAHPS between White and other racial and ethnic groups.

We implemented sensitivity analyses to test the robustness of our study. We created binary variables for each of these 6 measures, which equaled 1 if the ACO CAHPS (ACO1-ACO6) measure was above the 75th percentile (ie, the highest [best] top 25th percentile). Furthermore, we created a composite ACO CAHPS of 1 if any of the 6 ACOs exceeded the 25th highest percentile. We reported these findings in the eFigure and eTable in the Supplement. Results were consistent with our main findings.

STATA statistical software version 17 MP4 (StataCorp) was used to analyze the data. Statistical analysis was done with 2-sided hypothesis tests, including χ^2^ tests for categorical variables, analysis of variance tests for continuous variables, *t* tests for the mean differences by each race and ethnicity group, and ordinary least square (OLS) regression to estimate the variation of ACO CAHPS measures by race and ethnicity. The statistical significance was set at *P* < .05. The analysis was implemented from November 1, 2021, to July 29, 2022.

## Results

The final sample included 568 368 beneficiaries (347 783 female patients [61.2%]; 38 030 African American patients [6.69%], 6258 Asian patients [1.10%], 18 231 Hispanic patients [3.21%], and 505 849 White patients [89.0%]; mean [SD] age, 82.17 [7.95] years). Rates of diabetes were substantially higher among African American or Black, Asian and Hispanic populations than White counterparts. Other racial groups were more likely to live in urban areas than White patients. African American or Black and Hispanic patients with ADRD were also more likely to live in areas with lower education, higher poverty rates, and mental health shortage areas. Full demographic information is available in Table 1.

**Table 1.** Characteristics of Medicare Fee-for-Service Beneficiaries Enrolled in Medicare Shared Savings Program Accountable Care Organization Aged 65 Years and Older With Alzheimer Disease and Related Dementias by Race and Ethnicity

Characteristic^c^	CAHPS score, mean (SD)^a^^,^^b^				*P* value^d^
	African American or Black (n = 38 030)	Asian (n = 6258)	Hispanic (n = 18 231)	Non-Hispanic White (n = 505 849)	
Age, y					<.001
65-74	0.22 (0.41)	0.20 (0.40)	0.21 (0.41)	0.15 (0.36)	
75-84	0.37 (0.48)	0.35 (0.48)	0.37 (0.48)	0.35 (0.48)	
≥85	0.33 (0.47)	0.37 (0.48)	0.35 (0.48)	0.42 (0.49)	
Sex					<.001
Male	0.36 (0.48)	0.4 (0.49)	0.4 (0.49)	0.39 (0.49)	
Female	0.64 (0.48)	0.6 (0.49)	0.6 (0.49)	0.61 (0.49)	
Chronic conditions					
Acute myocardial infarction	0.01 (0.11)	0.01 (0.09)	0.01 (0.11)	0.01 (0.1)	<.001
Asthma	0.06 (0.24)	0.05 (0.22)	0.06 (0.24)	0.05 (0.21)	<.001
Atrial fibrillation	0.08 (0.27)	0.09 (0.29)	0.10 (0.3)	0.17 (0.37)	<.001
Heart failure	0.27 (0.44)	0.17 (0.38)	0.23 (0.42)	0.24 (0.43)	<.001
Chronic obstructive pulmonary disease	0.13 (0.33)	0.08 (0.28)	0.12 (0.32)	0.16 (0.36)	<.001
Depression	0.22 (0.42)	0.24 (0.43)	0.3 (0.46)	0.33 (0.47)	<.001
Diabetes	0.47 (0.5)	0.42 (0.49)	0.47 (0.5)	0.29 (0.45)	<.001
Hyperlipidemia	0.58 (0.49)	0.58 (0.49)	0.61 (0.49)	0.57 (0.5)	<.001
Hypertension	0.85 (0.36)	0.71 (0.45)	0.76 (0.42)	0.74 (0.44)	<.001
Stroke/transient ischemic attack	0.11 (0.31)	0.09 (0.28)	0.09 (0.29)	0.09 (0.29)	<.001
Rural vs urban status					<.001
Rural	0.03 (0.17)	0.01 (0.09)	0.07 (0.26)	0.03 (0.18)	
Nonmetropolitan	0.07 (0.25)	0.03 (0.16)	0.06 (0.23)	0.15 (0.35)	
Urban	0.90 (0.30)	0.96 (0.19)	0.87 (0.33)	0.82 (0.38)	
Zip-code level, %					
High school degree and higher	86.00 (7.55)	90.35 (7.60)	83.71 (11.78)	90.28 (6.38)	<.001
Poverty	18.62 (10.45)	10.52 (6.93)	15.77 (9.68)	11.62 (7.13)	<.001
Black	41.06 (28.83)	9.57 (12.54)	9.57 (13.01)	8.65 (12.1)	<.001
Whole county mental health shortage area	0.17 (0.37)	0.09 (0.29)	0.24 (0.43)	0.26 (0.44)	<.001
Area Deprivation Index >50th percentile	0.63 (0.48)	0.24 (0.42)	0.56 (0.50)	0.50 (0.50)	<.001

^a^ Sample size: Our study focused on patients who had a diagnosis of Alzheimer disease and related dementias, using the measure of the CMS Chronic Conditions Data Warehouse. The sample was limited to community-based fee-for-service (at least 12 months) beneficiaries aged 65 years and older. Medicare Advantage and dual-eligible beneficiaries were excluded from the analysis (1 654 646 beneficiaries). Our study focused on African American or Black, Asian, Hispanic, and White patients (1 630 414 beneficiaries). Patients were linked to the ACO beneficiary level file shared savings program (591 920 beneficiaries). Our study focused on the observations with all the 6 ACO CAHPS measures (576 583 beneficiaries). The resulting sample for the analysis included 568 368 beneficiaries after dropping the observations with missing measures of Area Deprivation Index.

^b^ All the variables from age to urban are dummy variables.

^c^ We used the 2017 Centers for Medicare & Medicaid Services (CMS) Medicare inpatient claims data and Medicare Beneficiary Summary File (MBSF). We further linked the CMS MBSF with the 2017 Medicare Shared Savings Program (MSSP) Accountable Care Organization (ACO) data to capture ACO characteristics. We used the ACO Consumer Assessment of Healthcare Providers and Systems (CAHPS) evaluations reported by the CMS 2017 ACO Quality Reporting Documentation.^18^

^d^ Tests were used to test the mean differences of 4 racial and ethnic groups. χ^2^ tests were used for categorical variables. Analysis of variance tests were used for continuous variables.

The comparison of the ACO CAHPS scores by race and ethnicity among Medicare fee-for-service beneficiaries aged 65 years and older are presented in Table 2. Results showed that White patients reported higher ACO CAHPS total score, and higher CAHPS scores on receiving timely care (ACO1), provider communication (ACO2), patients’ rating (ACO3), and access to specialists (ACO4), compared with African American or Black, Asian, and Hispanic patients with ADRD. Compared with White beneficiaries, Hispanic beneficiaries reported higher scores on health promotion (ACO5), and Asian and Hispanic beneficiaries reported higher scores on shared decision-making (ACO6).

**Table 2.** ACO CAHPS Scores Among Medicare Fee-for-Service Beneficiaries Aged 65 Years and Older With Alzheimer Disease and Related Dementias by Race and Ethnicity

CAHPS Category^b^	African American or Black (n = 38 030)^a^		Asian (n = 6258)^a^		Hispanic (n = 18 231)^a^		Non-Hispanic White [n = 505 849], mean (SD)
	Mean (SD)	*P* value^c^	Mean (SD)	*P* value^c^	Mean (SD)	*P* value^c^	
ACO CAHPS sum	485.81 (9.32)	<.001	486.18 (9.55)	<.001	486.76 (9.34)	<.001	487.51 (9.19)
CAHPS: Getting Timely Care, Appointments, and Information (ACO1)	79.71 (3.48)	<.001	80.24 (3.47)	<.001	79.4 (3.46)	<.001	80.65 (3.38)
CAHPS: How Well Your Providers Communicate (ACO2)	92.94 (1.62)	<.001	92.91 (1.6)	<.001	92.95 (1.59)	<.001	93.09 (1.45)
CAHPS: Patients’ Rating of Provider (ACO3)	92.18 (1.72)	<.001	92.22 (1.61)	.002	92.18 (1.84)	<.001	92.28 (1.54)
CAHPS: Access to Specialists (ACO4)	83.33 (2.08)	<.001	82.76 (2.4)	<.001	83.32 (2.28)	<.001	83.39 (2.15)
CAHPS: Health Promotion and Education (ACO5)	62.75 (3.31)	<.001	61.94 (3.3)	<.001	62.77 (3.23)	<.001	62.21 (3.55)
CAHPS: Shared Decision-Making (ACO6)	74.90 (2.47)	<.001	76.12 (2.31)	<.001	76.14 (2.39)	<.001	75.90 (2.28)

Abbreviations: ACO, Accountable Care Organizations; CAHPS, Consumer Assessment of Healthcare Providers and Systems.

^a^ Our study focused on patients who had a diagnosis of ADRD, using the measure of the CMS Chronic Conditions Data Warehouse. The sample was limited to community-based fee-for-service (at least 12 months) beneficiaries 65 years of age and older. Medicare Advantage and dual-eligible beneficiaries were excluded from the analysis. Patients were linked to the ACO beneficiary level file shared savings program.

^b^ We used the 2017 Centers for Medicare and Medicaid Services Medicare inpatient claims data and Medicare Beneficiary Summary File (MBSF). We further linked the CMS MBSF with the 2017 Medicare Shared Savings Program (MSSP) ACO data to capture ACO characteristics. We used the ACO CAHPS evaluations reported by the CMS 2017 ACO Quality Reporting Documentation.^18^ ACOs were continuous measures with possible ranges from 0 to 100. In our study sample, the range of ACO1 was 60.98 to 88.71, the range of ACO2 was 71.85 to 96.11, the range of ACO3 was 74.3 to 95.88, the range of ACO4 was 72.17 to 89.35, the range of ACO5 was 48.25 to 72.82, the range of ACO6 was 67.56 to 82.69. The range of the ACO CAHPS sum was 406.67 to 510.94.

^c^ *t* tests were used to test the mean differences by each race and ethnicity group using Non-Hispanic White as the reference group.

After controlling for characteristics of beneficiary demographic and health indicators, model 1 showed significant racial and ethnic disparities in enrolling in ACOs with high ACO CAHPS scores (Table 3). Disparities between African American or Black patients (coefficient = -1.051; 95% CI, −1.15 to -0.95; *P* < .001) and White patients (coefficient = −0.414; 95% CI, −0.623 to −0.205; *P* < .001) and Asian patients (coefficient = −0.099; 95% CI, −0.229 to 0.032; *P* = .14) and White patients remain after controlling for SDOH and applying state-fixed effects (model 2). Disparities of the CAHPS total score between Hispanic and White individuals became insignificant. Results also showed that compared with urban residents, residents in rural and nonmetropolitan areas were less likely to enroll in ACOs with high CAHPS scores. Residents in areas with low education, higher poverty rate, a higher African American or Black population, and mental health shortage areas were also less likely to enroll in ACOs with high CAHPS scores.

**Table 3.** Regression Results of Accountable Care Organizations Consumer Assessment of Healthcare Providers and Systems Total Score

Characteristic	Model 1: Beneficiary-level characteristics^a^		Model 2: Model 1 plus community characteristics, state fixed effect^a^	
	Coefficient (95%CI)	*P* value	Coefficient (95%CI)	*P* value
Race and ethnicity				
African American or Black	−1.603 (−1.700 to −1.505)	<.001	−1.051 (−1.153 to −0.949)	<.001
Asian	−1.323 (−1.553 to −1.094)	<.001	−0.414 (−0.623 to −0.205)	<.001
Hispanic	−0.715 (−0.851 to −0.578)	<.001	−0.099 (−0.229 to 0.032)	.14
Non-Hispanic White	1 [Reference]		1 [Reference]	
Age, y				
65-74	1 [Reference]		1 [Reference]	
75-84	0.023 (−0.041 to 0.087)	.48	0.031 (−0.027 to 0.089)	.29
≥85	0.243 (0.179 to 0.306)	<.001	0.081 (0.023 to 0.139)	.006
Female	0.008 (−0.042 to 0.058)	.75	−0.009 (−0.054 to 0.037)	.71
Acute myocardial infarction	−0.179 (−0.414 to 0.056)	.14	−0.114 (−0.327 to 0.099)	.30
Asthma	0.265 (0.153 to 0.378)	<.001	0.258 (0.156 to 0.361)	<.001
Atrial fibrillation	0.350 (0.280 to 0.420)	<.001	0.216 (0.153 to 0.279)	<.001
Heart failure	−0.505 (−0.566 to −0.444)	<.001	−0.365 (−0.420 to −0.310)	<.001
Chronic obstructive pulmonary disease	−0.410 (−0.479 to −0.340)	<.001	−0.208 (−0.272 to −0.145)	<.001
Depression	0.150 (0.098 to 0.203)	<.001	0.015 (−0.033 to 0.062)	.54
Diabetes	−0.198 (−0.253 to −0.144)	<.001	−0.149 (−0.198 to −0.099)	<.001
Hyperlipidemia	0.553 (0.501 to 0.606)	<.001	0.366 (0.318 to 0.414)	<.001
Hypertension	−0.080 (−0.140 to −0.020)	.009	−0.125 (−0.180 to −0.071)	<.001
Stroke/transient ischemic attack	−0.025 (−0.109 to 0.059)	.56	−0.010 (−0.086 to 0.067)	.81
Urban	NA	NA	1 [Reference]	
Rural	NA	NA	−0.803 (−0.954 to −0.652)	<.001
Nonmetropolitan	NA	NA	−1.173 (−1.249 to −1.098)	<.001
Zip-code level (%)				
High school degree and higher	NA	NA	0.011 (0.006 to 0.015)	<.001
Poverty	NA	NA	−0.033 (−0.037 to −0.028)	<.001
Black	NA	NA	−0.021 (−0.023 to −0.019)	<.001
Whole county mental health shortage area	NA	NA	−1.256 (−1.322 to −1.189)	<.001
State fixed	NA	NA	1 [Reference]	
Constant	487.269 (487.194 to 487.343)	<.001	489.078 (488.556 to 489.599)	<.001

Abbreviation: NA, not applicable.

^a^ We used the ordinary least square regression to estimate the variation of ACO CAHPS measures by race. We first reported the regression results only controlling for beneficiaries’ demographic and health indicators (model 1). Then, we expanded model 1 by applying the state-fixed effect ordinary least square estimation and controlling for measures of SDOH (model 2).

Similarly, disparities were observed among individual CAHPS measures of getting timely care, provider communication, and patient’s rating (Table 4). African American or Black and Asian patients reported lower access to specialties, and African American or Black patients reported a lower rate of shared decision-making, compared with White patients. Compared with White beneficiaries, African American or Black and Hispanic patients reported higher CAHPS scores on health promotion and education, and Asian patients reported higher scores on shared decision-making. To facilitate the interpretation, in the eTable in the Supplement, we reported the results of the logistic regressions and tested the likelihood of enrolling in the ACOs with the top 25th percentile of each CAHPS score. Marginal effects were reported. Results were similar. Compared with White patients, African American or Black patients were less likely to enroll in the ACOs with the top scores of ACO1 through ACO6.

**Table 4.** Regression Results of Each Accountable Care Organizations Consumer Assessment of Healthcare Providers and Systems Score

Score category and race and ethnicity^a^	Coefficient (95% CI)	*P* value
Getting timely care, appointments, and information		
African American or Black	−0.36 (−0.40 to −0.33)	<.001
Asian	−0.08 (−0.16 to 0.00)	.04
Hispanic	−0.35 (−0.40 to −0.30)	<.001
Non-Hispanic White	1 [Reference]	NA
How well your providers communicate		
African American or Black	−0.18 (−0.19 to −0.16)	<.001
Asian	−0.16 (−0.19 to −0.12)	<.001
Hispanic	−0.11 (−0.13 to −0.09)	<.001
Non-Hispanic White	1 [Reference]	NA
Patients’ rating of provider		
African American or Black	−0.19 (−0.21 to −0.17)	<.001
Asian	−0.09 (−0.13 to −0.06)	<.001
Hispanic	−0.10 (−0.13 to −0.08)	<.001
Non-Hispanic White	1 [Reference]	NA
Access to specialists		
African American or Black	−0.17 (−0.19 to −0.15)	<.001
Asian	−0.26 (−0.31 to −0.21)	<.001
Hispanic	−0.01 (−0.04 to 0.02)	.53
Non-Hispanic White	1 [Reference]	NA
Health promotion and education		
African American or Black	0.07 (0.03 to 0.11)	<.001
Asian	−0.04 (−0.11 to 0.03)	.27
Hispanic	0.48 (0.43 to 0.52)	<.001
Non-Hispanic White	1 [Reference]	NA
Shared decision-making		
African American or Black	−0.22 (−0.25 to −0.20)	<.001
Asian	0.22 (0.17 to 0.27)	<.001
Hispanic	0.00 (−0.03 to 0.03)	.93
Non-Hispanic White	1 [Reference]	NA

Abbreviation: NA, not applicable

^a^ All other covariates were controlled, full model (model 2).

Results of the decomposition analyses estimated higher CAHPS total scores for White patients. Our empirical model explained a substantial part of racial and ethnic disparities (ie, 18% of African American or Black vs White, and 14% of Hispanic vs White) of CAHPS. Meanwhile, the results suggested the majority of disparities (82%-86%) could not be explained by our models. Among all the controlled variations, the ADI was the most substantial factor, which explained 13% and 11% of disparities between African American or Black vs White and Hispanic vs White patients with ADRD, respectively. Our model specification, however, could not explain disparities of CAHPS scores among Asian vs White patients with ADRD. Results showed that if all the controlled variables were the same between Asian and White patients, Asian patients were supposed to report higher CAHPS scores (ie, the negative explained score in Table 5).

**Table 5.** Decompose Accountable Care Organizations Consumer Assessment of Healthcare Providers and Systems (ACO CAHPS) Total Score by Race and Ethnicity According to the Full Model (Model 2)

Measure^a^	African American or Black predicted score^b^	*P* value^c^	Hispanic predicted score^b^	*P* value^c^	Asian predicted score^b^	*P* value^c^
Estimated score	485.81	<.001	486.77	<.001	486.18	<.001
Difference (with the predicted score of White = 487.5)^d^	−1.71	<.001	−0.75	<.001	−1.33	<.001
Total, %	17.60	<.001	13.79	<.001	−27.79	<.001
Explained individual factor Area Deprivation Index, %	12.65	<.001	10.29	<.001	−28.24	<.001

^a^ We used the 2017 Centers for Medicare and Medicaid Services (CMS) Medicare inpatient claims data and Medicare Beneficiary Summary File (MBSF). We further linked the CMS MBSF with the 2017 Medicare Shared Savings Program (MSSP) ACO data to capture ACO characteristics. We used the ACO CAHPS evaluations reported by the CMS 2017 ACO Quality Reporting Documentation.

^b^ Our study focused on patients who had a diagnosis of Alzheimer Disease or Related Dementias, using the measure of the CMS Chronic Conditions Data Warehouse. The sample was limited to community-based fee-for-service (at least 12 months) beneficiaries 65 years of age and older. Medicare Advantage and dual-eligible beneficiaries were excluded from the analysis. Patients were linked to the ACO beneficiary level file shared savings program.

^c^ We applied the Blinder-Oaxaca decomposition model to explain the factors associated with racial and ethnic disparities in CAHPS. For example, our empirical model explained 17.6% of White vs Black disparities in CAHPS. Among all the controlled variations, the Area Deprivation Index, a Social Determinant of Health indicator, was the most significant factor, which explained 12.65% of the observed racial disparities in CAHPS.

^d^ Non-Hispanic White was the reference group.

## Discussion

The findings of this cross-sectional study demonstrated significant racial and ethnic disparities in CAHPS scores. The results also showed that the ADI, an indicator of SDOH, explained 10% to 13% of disparities of ACO CAHPS between African American or Black vs White and Hispanic vs White patients with ADRD. Most of the racial and ethnic disparities, especially the disparities between Asian and White patients, could not be explained by beneficiaries’ demographic characteristics, coexisting health conditions, and the ADI. Our results support the literature that patients with ADRD belonging to racial and ethnic minority groups encountered worse quality uncoordinated health care, including a lack of well-timed access to primary care. Our assessment of ADI demonstrated the link between SDOH and existing health disparities. It is also possible that some disparities were tied to cultural preferences, availability of caregivers, biases, and other factors that were not measured using the existing data sets.

Racial and ethnic minority patients with ADRD reported lower CAHPS on getting timely care. Evidence suggests that minority populations are more likely to have later-stage diagnoses, experience delays in timely primary care, and lack access to coordinated care.^3,4,27^ Access to timely care may prevent disease progression. Results also suggested African American or Black patients reported lower CAHPS scores on communication, rating, access to specialties, and shared decision-making. Prompt communication between the person with dementia, their caregiver, and the team of health care clinicians is one of the critical factors in successful transitions in care.^28^ Taking a person-centered approach to care transitions has the potential to improve the quality of care provided—delivering person-centered care involves accounting for patients’ preferences while also considering the caregiver throughout the process.

Results showed encouraging findings on health promotion and education. African American or Black and Hispanic patients reported higher scores than White cohorts. Racial and ethnic minority groups, immigrants, persons with low socioeconomic status, and those with low self-reported health on average have a lower level of patient activation relative to their counterparts.^1^ We speculated that the ACO enrollment might have played a role in promoting health education, especially among African American or Black and Hispanic patients and their caregivers. It will be interesting to explore the different characteristics of health care clinicians that serve these minority patients. These clinicians might share their experience of effectively engaging minority patients with ADRD and their caregivers. We speculated that cultural competency training, language, and clinician-patient racial and cultural concordance might be critical.

Our study also found that SDOH ADI was associated with ACO CAHPS, indicating the need for targeted approaches to address SDOH. Several SDOH are associated with cognition and ADRD diagnosis, such as low socioeconomic status, low levels of education, economically disadvantaged regions with limited physical resources, and food security, among other factors.^3^^,^^29^ Results of our study suggested that ACOs aimed at improving access to necessary, timely care may help decrease existing disparities. Resources can be directed toward SDOH to improve the well-being of older adults. Our findings were also consistent with the structural racism and discrimination (SRD) literature and supported the framework of SRD patients with ADRD^30,31,32,33,34^. The components of SRD, such as institutional racism, systemic barriers, and other health determinants, are key factors to target to decrease disparities. In addition, the present study is the first, to our knowledge, to measure the SRD at the intermediary level (ie, physician-patient interaction) for the ADRD population.

Our findings suggested that there are substantial rural and urban disparities in enrollment of beneficiaries in ACOs with high CAHPS. Often, rural regions have fewer resources than urban areas. Variations in the availability of health care services between rural and urban regions may affect timely access to care. One study^35^ found that older Medicare fee-for-servicebeneficiaries in rural areas are potentially underdiagnosed with ADRD and receive delayed diagnosis.

Given our growing diverse population of older adults, it is imperative that future research reflects their care experiences and needs. For example, given the lack of research on older Asian American adults, we are limited in our ability to fully explain the disparities in our study. From the literature on the aging Asian immigrants receiving care for depression, we speculated that implementing a patient-centered model in decision-making could lead to the positive engagement of the individual.

### Limitations

Our study had several limitations. First, our model used multilevel controls according to the National Institute on Minority Health and Health Disparities framework but still might have missed other variables, such as the severity of the diseases, and other factors missing in the claims data, such as measures reflecting preferences, caregiver availability, and burdens, among others. Second, our study focused on MSSP ACO enrollees. Results should not be applied to the general aging population. Although the study population was homogeneous, we were able to observe significant variation among individuals belonging to different racial and ethnic groups. According to the literature, ACO enrollment might be associated with racial disparities, and White individuals were overrepresented. It is also possible that people not enrolled in an ACO experience a lower quality of care and worse disparities. Future studies may improve the data collection of the CAHPS measures to facilitate the assessment of the aging population. Additionally, our study documented CAHPS variation among patients with ADRD of different races and ethnicities. Future studies may further investigate modifiable factors that can be used to reduce observed disparities. It is a teachable moment to promote health equity and to use the opportunities of ongoing policy initiatives such as reimbursement and improving health infrastructure.

## Conclusions

The burden of ADRD in the US will become increasingly high as the aging population grows and racial and ethnic diversity increases. CAHPS measures have been used widely to measure patient-centered care. It is also a proxy measure of intermediary determinants (ie, the interpersonal experience) of SRD. Our results demonstrated significant variations in CAHPS by race and ethnicity among patients with ADRD. Results suggested the urgent need to understand the factors that contributed to such disparities. Policy initiatives targeting the structural factors, such as payment reform and care coordination, might be useful to improve CAHPS for racial and ethnic minority patients with ADRD, a population that has faced SRD during their life courses.

## Notes

Supplement. **eFigure.** ACO CAHPS Top 25th Percentile by Race and Ethnicity **eTable.** Regression Results of ACO CAHPS Top 25th Percentile, Marginal Effects Were Reported
