# Supplementary material for: Consumer Assessment of Healthcare Providers and Systems Among Racial and Ethnic Minority Patients With Alzheimer Disease and Related Dementias
Source: JAMA Netw Open. 2022 Sep 27;5(9):e2233436. doi: 10.1001/jamanetworkopen.2022.33436 (PMC9516284; doi:10.1001/jamanetworkopen.2022.33436)
Supplement: Supplement. — eFigure. ACO CAHPS Top 25th Percentile by Race and Ethnicity eTable. Regression Results of ACO CAHPS Top 25th Percentile, Marginal Effects Were Reported [file jamanetwopen-e2233436-s001.pdf]

Supplemental Online Content

Albaroudi A, Chen J. Consumer Assessment Of Healthcare Providers And Systems among racial and ethnic minority patients with Alzheimer disease and related dementias. *JAMA Netw Open*. 2022;5(9):e2233436.
doi:10.1001/jamanetworkopen.2022.33436

eFigure. ACO CAHPS Top 25th Percentile by Race and Ethnicity
eTable. Regression Results of ACO CAHPS Top 25th Percentile, Marginal Effects Were Reported

This supplemental material has been provided by the authors to give readers additional information about their work.

© 2022 Albaroudi A et al. *JAMA Network Open.*

eFigure. ACO CAHPS Top 25th Percentile by Race and Ethnicity

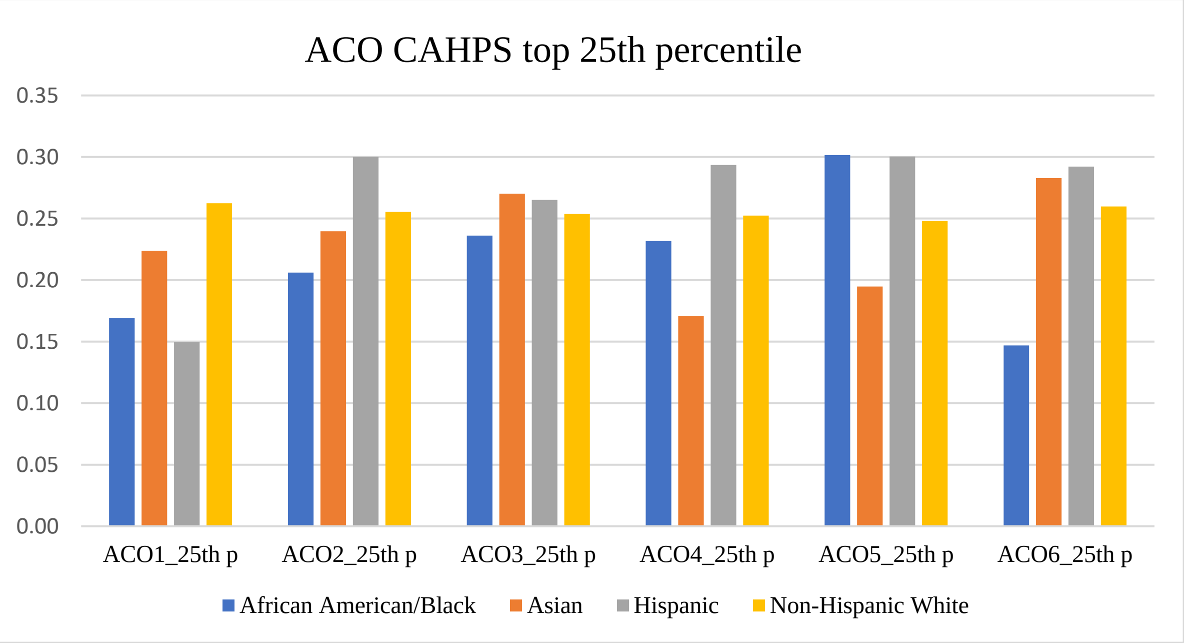

eTable. Regression Results of ACO CAHPS Top 25th Percentile, Marginal Effects Were Reported

	**Any CAHPS individual score is in the top 25th percentile**			
	ME	95% CI		p
White	reference			
Black	-0.024	-0.029	-0.019	<0.001
Asian	0.005	-0.005	0.016	0.33
Hispanic	0.001	-0.006	0.008	0.76
	**Getting Timely Care, Appointments, and Information, top 25th percentile**			
	ME	95% CI		p
White	reference			
Black	-0.030	-0.035	-0.025	<0.001
Asian	0.004	-0.006	0.013	0.44
Hispanic	-0.035	-0.042	-0.028	<0.001
	**How Well Your Providers Communicate, top 25th percentile**			
	ME	95% CI		p
White	reference			
Black	-0.027	-0.032	-0.022	<0.001
Asian	-0.015	-0.025	-0.005	0.004
Hispanic	-0.021	-0.027	-0.015	<0.001
	**Patients' Rating of Provider top 25th percentile**			
	ME	95% CI		p
White	reference			

© 2022 Albaroudi A et al. *JAMA Network Open.*

Black	-0.024	-0.029	-0.019	<0.001
Asian	0.005	-0.005	0.014	0.32
Hispanic	0.000	-0.007	0.006	0.88
	**Access to Specialists top 25th percentile**			
	ME	95% CI		p
White	reference			
Black	-0.026	-0.031	-0.021	<0.001
Asian	-0.041	-0.052	-0.029	<0.001
Hispanic	0.037	0.031	0.043	<0.001
	**Health Promotion and Education top 25th percentile**			
	ME	95% CI		p
White	reference			
Black	0.010	0.006	0.014	<0.001
Asian	-0.007	-0.017	0.004	0.20
Hispanic	0.075	0.069	0.080	<0.001
	**Shared Decision Making top 25th percentile**			
	ME	95% CI		p
White	reference			
Black	-0.020	-0.025	-0.015	<0.001
Asian	0.028	0.019	0.037	<0.001
Hispanic	0.017	0.011	0.023	<0.001

© 2022 Albaroudi A et al. *JAMA Network Open.*
